# Molecular correlates of immune cytolytic subgroups in colorectal cancer by integrated genomics analysis

**DOI:** 10.1093/narcan/zcab005

**Published:** 2021-03-02

**Authors:** Constantinos Roufas, Ilias Georgakopoulos-Soares, Apostolos Zaravinos

**Affiliations:** Department of Life Sciences, School of Sciences, European University Cyprus, 1516 Nicosia, Cyprus; Department of Bioengineering and Therapeutic Sciences, University of California, San Francisco, CA 94158, USA; Institute for Human Genetics, University of California, San Francisco, CA 94158, USA; Department of Basic Medical Sciences, College of Medicine, Member of QU Health, Qatar University, 2713 Doha, Qatar

## Abstract

Although immune checkpoint inhibition (ICI) has shown promising results in metastatic dMMR/MSI-H colorectal cancer (CRC), the majority of pMMR/MSS patients do not respond to such therapies. To systematically evaluate the determinants of immune response in CRC, we explored whether patients with diverse levels of immune cytolytic activity (CYT) have different patterns of chromothripsis and kataegis. Analysis of CRC genomic data from the TCGA, indicated an excess of chromothriptic clusters among CYT-low colon adenocarcinomas, affecting known cancer drivers (*APC, KRAS, BRAF, TP53* and *FBXW7*), immune checkpoints (*CD274, PDCD1LG2, IDO1/2* and *LAG3*) and immune-related genes (*ENTPD1, PRF1, NKG7, FAS, GZMA/B/H/K* and *CD73*). CYT-high tumors were characterized by hypermutation, enrichment in APOBEC-associated mutations and kataegis events, as well as APOBEC activation. We also assessed differences in the most prevalent mutational signatures (SBS15, SBS20, SBS54 and DBS2) across cytolytic subgroups. Regarding the composition of immune cells in the tumor milieu, we found enrichment of M1 macrophages, CD8+ T cells and Tregs, as well as higher CD8+ T-cells/Tregs ratio among CYT-high tumors. CYT-high patients had higher immunophenoscores, which is predictive of their responsiveness if they were to be treated with anti-PD-1 alone or in combination with anti-CTLA-4 drugs. These results could have implications for patient responsiveness to immune checkpoint inhibitors.

## INTRODUCTION

Colorectal cancer (CRC) ranks in the third position of incidence and mortality with ∼1.4 million cases being diagnosed worldwide each year (1). The mutational landscape for the majority of the tumors (∼85%) consists of chromosomal instability, loss of heterozygosity, chromosomal amplifications and translocations. The remaining 15% of tumors are usually characterized by a defective DNA mismatch repair system (dMMR), due to mutations or epigenetic silencing of MMR genes, such as MSH2 and MLH1 (2,3). These usually result in microsatellite instability (MSI) and eventually to the accumulation of mutations (4). The buildup of DNA mutations facilitates the formation of immunogenic cancer neoepitopes, i.e. tumor-mutated peptides which attract various immune-related cells within the tumor micro-environment (TME), including neutrophils, dendritic cells (DCs), macrophages, natural killer (NK) cells, T cells and B cells (5,6).

Being quite heterogeneous, the disease can be classified into four different consensus molecular subtypes (CMS1, MSI Immune, 14%; CMS2, Canonical, 37%; CMS3, Metabolic, 13%; and CMS4, Mesenchymal, 23%) (7,8). CMS1 tumors have a similar to the MSI-like subgroup with healthy tumor-infiltrating lymphocytes (TILs). CMS2 is characterized by activation of the Wnt pathway and by a cold TME. WNT inhibition is expected to foster an anti-tumoral immune response in these tumors (9). CMS3 generally depends on the activation of the interconnected MAPK/PI3K signaling pathways; whereas, CMS4 is mainly characterized by activation of the TGFβ signaling pathway (7). Immunologically, the TME in CRC is very heterogeneous, depending on the CMS. Out of the four subtypes, CMS1 tumors are characterized by increased immune infiltration and immune activation (8,10). The classification for different immunological subtypes in CRC can predict response to immunotherapy and enhance antitumor activity (11,12). The TME is characterized by different cell types including multiple immune cells, and its composition may predict the prognosis of patients, as well as their response to therapies (7,13). As the TME promotes cancer progression (14), and abnormalities in it can interrupt immunotherapy, its understanding is of major importance in tackling the disease. In addition, the level of cytotoxic T cells (CTLs) affects patient survival (15,16).

The antitumoral immune cytolytic activity (CYT), calculated from the mRNA expression of granzyme A (*GZMA*) and perforin 1 (*PRF1*), is a relatively new indicator of cancer immunity (12,17,18). Perforin is a pore-forming toxin, whereas granzymes (GZMA, GZMB, GZMH, GZMK and GZMM) are serine proteases which are stored within cytotoxic granules of CTL and NK cells. Once overexpressed, these two enzymes mediate the apoptosis of cancer cells in a cooperative manner (19).

The standard treatment options vary from surgical removal alone or surgical removal followed by adjuvant 5-fluoruracil-based chemotherapy or targeted therapy, depending on the stage of the disease (20). Immune checkpoint blockade has revolutionized cancer treatment stimulating an anticancer response, mainly through the selective targeting of the PD-1/PD-L1 axis and the CTLA-4 receptor (21–23). Inhibitors against other immune-regulating molecules, such as LAG-3, are also being developed (22). Recently, immune checkpoint inhibitors have demonstrated impressive activity in dMMR/MSI-H metastatic CRCs (24,25) or in hypermutated tumors harboring alterations in DNA polymerases δ (POLD1) or ϵ (POLE) (26,27). However, these tumors constitute only a minority. Thus, the greatest challenge is to induce the majority of tumors, which have a proficient MMR (pMMR) to exhibit immunologic properties and/or responsiveness to immunotherapy, similar to dMMR/MSI-H cancers (28,29). Therefore, the determinants of immune response in CRC need a better understanding.

Somatic mutations in cancer genomes are caused by multiple mutational processes, each of which generates a characteristic mutational signature. Although the mutational processes contributing to the development of different types of cancer have been extensively studied during the last decade (30–32), their association with the tumor's immune profile have remained partially understood. Thus, mutational signature analysis could help us further stratify CRC patients and inform us on differences in response to immunotherapy.

Recent evidence suggests that 2–3% of all cancers contain very complex rearrangements in their genome, associated with two copy number (CN) states (33,34). These events usually involve complete chromosomes or chromosome arms and result from massive chromosomal fragmentation occurring in one catastrophic event, also known as chromothripsis (33,34). Although chromothripsis is a common event in CRC (35), its impact on immune checkpoint genes and genes associated with CYT is not known.

Another complex event recently recognized in the genome of cancers, is kataegis. The term refers to a pattern of clustered hypermutations, mainly C > G and/or C > T mutations, identified in the genome of some cancers, in which a large number of highly patterned mutations occur sequentially in a small region of DNA (36). Kataegis has been associated with promiscuous activities of the ‘activation-induced cytidine deaminase’ (AID), ‘apolipoprotein B mRNA editing enzymes, catalytic polypeptide-like’ (APOBEC) and genomic rearrangements in B cell lymphomas (37) and other tumors (38,39). The AID/APOBEC family contains members that can deaminate cytidine in RNA or DNA and exhibit diverse physiological functions (40,41). Compared to chromothripsis, kataegis is more common in cancer genomes and causes mutational bursts rather than accumulating in a step-wise fashion (42–44).

Herein, we investigated whether colorectal tumors belonging to different subgroups of immune CYT have diverse patterns of chromothripsis and kataegis. We also assessed differences in the most prevalent mutational signatures across the cytolytic subgroups of tumors, and explored the proportion of different immune cell types and cancer neoantigens in them. Finally, we used immunophenoscores as a proxy providing information regarding the potential responsiveness to immune checkpoint inhibition therapy, using as a criterion their immune CYT index.

## MATERIALS AND METHODS

### Genomic data extraction

We extracted data from two TCGA datasets, the colon adenocarcinoma (COAD, *n* = 461) and rectum adenocarcinoma (READ, *n* = 172). Gene expression ‘level 3’ mRNA-Seq data for tumor and normal samples, Mutation Annotation Format (MAF) files, CNV files, along with the corresponding patient clinical information, were all extracted using the GDC data portal (https://portal.gdc.cancer.gov/). We pre-processed data using the Apache Spark program in Python and further analyzed them using R, as described before (12). We used iCoMut (v.0.21) for FireBrowse to perform comutation analysis. We categorized and discriminated hypermutated (>34 mutations/Mb) from non-hypermutated (<34 mutations/Mb) samples, as described previously (45).

### Determination of immune cytolytic activity

We determined each patient's immune CYT levels, according to the expression of G*ZMA* and *PRF1* (12,17,18,46). Cancer patients were separated into two immune cytolytic subgroups, each representing the upper and lower quartiles of the cytolytic index, as previously reported by our group (12). All subsequent comparisons were made between these two cytolytic subgroups of colon and rectal cancers. All *P*-values were False Discovery Rate (FDR)-adjusted.

### Calculation of somatic mutations and copy number alterations (SCNA)

The MAF files were processed using Maftools (47) and the presence of somatic mutations between the two cytolytic subsets was performed using MutSig (v1.3.01) (48,49). We calculated the mutation rate as the number of somatic mutations per million bases per patient. CN gains or losses were recognized using GISTIC2 (50). CN gains were defined as genes showing log_2_(CN ratio) ≥ 0.1 and CN losses were defined as genes showing log_2_(CN ratio) ≤ −0.1. Amplified or deleted genomic regions within each CYT subgroup with an FDR < 0.25 were considered significant. We used adjusted *P*-values to account for multiple testing.

### Tumor heterogeneity and MATH scores within cytolytic subsets of CRC

We inferred intra-tumoral genomic heterogeneity by clustering the variant allele frequencies as measured by the width of its distribution. A mutant-allele tumor heterogeneity (MATH) score was assigned to each tumor (51). No differences in the MATH scores were detected between diverse cytolytic subsets in COAD or READ tumors.

### Mutational signatures within cytolytic subsets of CRC

We extracted single base substitutions (SBS) using 96 different contexts, considering not only the mutated base, but also the bases immediately 5′ and 3′ (30,52). We also identified doublet base substitutions (DBS), which are generated after the concurrent modification of two consecutive nucleotide bases. Once extracted, DBSs were linked to each of 78 known strand-agnostic DBS mutation types (30). Mutational signatures were analyzed using SigProfiler's MatrixGenerator and Extractor bioinformatic tools in Python (53). MAF files were used as input and the GRCh38 as the reference genome. The extracted signatures were then compared against 72 known SBS signatures and 11 known DBS signatures from the Catalogue of Somatic Mutations in Cancer (COSMIC v3.1). The contribution of each signature was calculated for each cytolytic subgroup in COAD and READ tumors and statistical significance was calculated with the Mann–Whitney U test, the *P*-values of which were Bonferroni-corrected. Tumors were hierarchically clustered according to their percentage of signature contribution, using seaborn ‘clustermap’ function.

### Rainfall plots within cytolytic subsets of CRC

The existence of localized hyper-mutations, or kataegis, forming unique mutation signatures has been described in the genomes of several cancers (36,39,52). We defined as kataegic regions in each patient's genome, those containing ≥6 sequential mutations with an average inter-mutation distance of ≤1000 bp (52), and compared them between the two cytolytic subgroups.

### Estimation of APOBEC-enrichment

We estimated APOBEC enrichment as previously described (54). Briefly, enrichment of C>T mutations occurring within tCw motifs, where ‘w’ corresponds to either adenine (A) or thymine (T), over all of the C>T mutations in each sample, was compared to the background C’s and tCw's occurring within 20 bp of the mutated bases, using the formula: n_tCw_ ∗ background_C_/n_C_ ∗ background_tCw_. We used the one-sided Fisher's exact test to statistically evaluate the enrichment score of APOBEC signature mutations (54). We compared the ratio of the number of C-to-T or C-to-G and G-to-A or G-to-C substitutions detected inside and outside of the APOBEC target motif (tCw) to an analogous ratio for all C’s and C’s residing inside and outside of the tCw motif within a sample fraction of the genome.

Differences in the mutational patterns between APOBEC-enriched and non-APOBEC enriched tumors, were assessed by taking APOBEC enrichment scores and classifying tumor samples into ‘APOBEC-enriched’ and ‘non-APOBEC enriched’ groups (54). The Benjamini–Hochberg method was used to correct *P*-values and only corrected *q*-values of <0.05 were considered significant.

### Chromothripsis in cytolytic subgroups in CRC

Chromothriptic events in the two cytolytic subsets in CRC were investigated using CTLPScanner (55). In brief, we downloaded level 3 segmentation data of single nucleotide polymorphism arrays from the two TCGA datasets and analyzed them using a circular binary segmentation algorithm (56) (hg38). To detect chromothripsis or chromothripsis-like regions, we used the following criteria: CN change ≥20 times, likelihood ratio (LR) (log10) ≥ 8, minimum segment size = 10 kb, and signal distance between adjacent segments = 0.3. Shattered chromosomal regions were visualized based on the signal value for genomic gains (≥0.15) or losses (≤−0.15). We lastly annotated chromothripsis-located genes with COSMIC release v92 (57).

### Immune cell fraction analysis

The Cancer Immunome Atlas (TCIA, https://tcia.at/) (58) was used to estimate cell type fractions in CYT-high or low cancers in the TCGA-COAD and TCGA-READ databases.

### Detection of cancer neoantigens and immunophenoscores

We mined information regarding the cellular composition of cancer neoantigens per Mb in the two cytolytic subsets in each CRC, from TCIA (58). We calculated each patient's immunophenoscore (IPS) within CYT-high and low subsets (58,59) and constructed immunophenograms to visualize each tumor's different immunophenotypes. The IPS scores (0–10 range), were based on the expression of MHC molecules, CTLA-4, LAG3, TIGIT, HAVCR2, CD274, PCDC1LG2, CD27, ICOS, IDO1 (immunomodulators), activated CD8+ and CD4+ T cells (effector cells), effector memory CD8+ and CD4+ T cells (Tem) and regulatory T cells (Tregs) or myeloid-derived suppressor cells (immune suppressor cells) (59).

## RESULTS

### CYT-high colon tumors are hypermutated and enriched in kataegis

**Figure 1.**
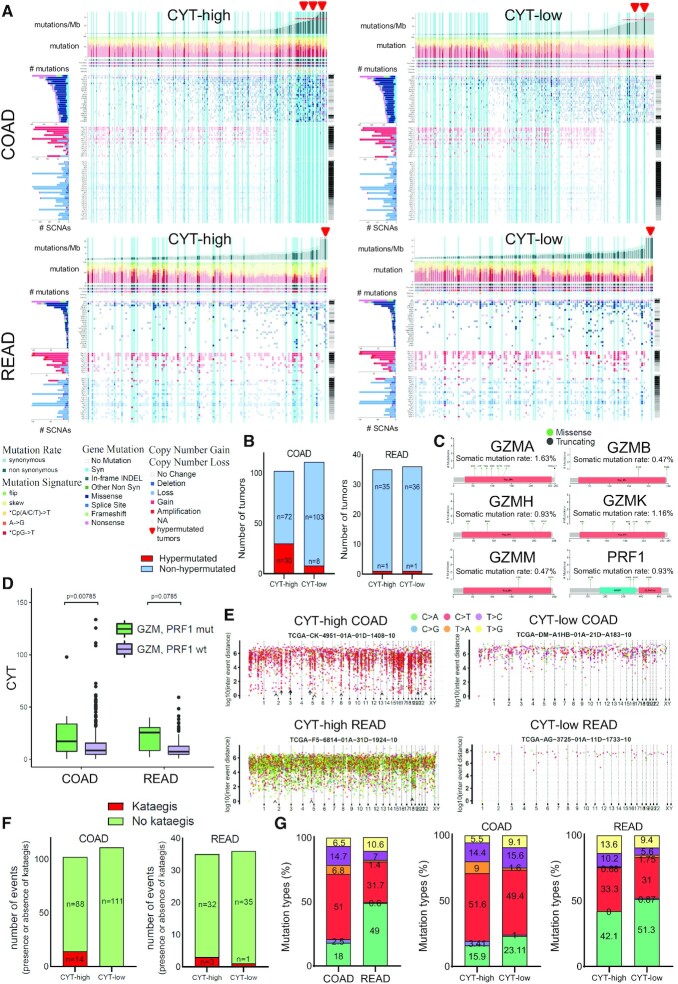
(**A**) Integrated plots of clinical and molecular features for all COAD (A) and READ samples, ordered by mutation rate (hypermutated, >34 mutations/Mb; non-hypermutated, <34 mutations/Mb). From top to bottom, panels indicate the frequency of mutations/Mb; the mutational signatures, indicating type of substitution; patient age, vital status, sex, histology and ethnicity; number of CNVs. The vertical light blue lines highlight the immune cytolytic high (or low, respectively) tumors in each co-mutation plot. Hypermutated tumors (>34 mutations/Mb) are indicated in red arrowheads. (**B**) In COAD, 30/102 (29.41%) CYT-high and only 8/111 (7.2%) CYT-low tumors were hypermutated. The hypermutation rate was similar between the two cytolytic subgroups in READ (∼2.8%). (**C**) Lollipop plots highlighting somatic point mutations (missense and truncating) in the domains of GZMA (A55D, L72F, T86A, K98N, Q119H, N127S), GZMB (E174K, R246S), GZMH (A36V, R84W, P205S, G214C), GZMK (S34L, I117N, S171*, T174N, K211N), GZMM (L168I, K251N) and PRF1 (A116S, D340G, A359T, G444C) proteins and their corresponding rates. Tryp_SPc, Trypsin-like serine protease; MACPF, Membrane Attack Complex/Perforin; C2_Perforin, C2 domain in the C-terminus of Perforin. (**D**) COAD and READ tumors harboring somatic mutations in granzymes (*GZMA, GZMB, GZMH, GZMK* and *GZMM*) and perforin (*PRF1*) genes had significantly higher CYT index compared to the wild-type (wt) tumors. (**E**) Kataegis (clustered hypermutations) was more evident across CYT-high CRCs. The representative rainfall plots display the intermutational distance across the genome of two cytolytic-distinct subgroups in colon (COAD) and rectum (READ) adenocarcinoma. The chromosomal domains with kataegis in the CYT-high tumors are pointed with arrows and arrowheads. (**F**) Number of events (presence or absence of kataegis) detected in the two cytolytic subsets in COAD and READ tumors. The majority of COAD tumors having kataegic sites (14/20, 70%) were CYT-high, whereas none of them was CYT-low. In READ, only 3 CYT-high and 1 CYT-low tumors had kataegis events, respectively. (**G**) Rates of the different mutation types (%) across COAD and READ tumors. The majority of mutations were C>T (50%) in COAD, and C>A (49%) in READ. Differences in the rates of the mutation types were noted between CYT-high and CYT-low tumors.

We initially performed comutation analysis to visually display the mutations across patients in each cytolytic CRC cohort. We stratified tumors to hypermutated and non-hypermutated groups, according to their rate of synonymous and non-synonymous mutations, and noted that almost one third of CYT-high tumors were hypermutated (30/102, 29.41%), whereas the corresponding percentage in CYT-low tumors was much lower (8/111, 7.2%) (*P*-value < 0.001, Fisher's exact test). On the other hand, such differences could not be noticed in READ tumors (*n* = 1, *P* > 0.05) (Figure 1A and B; Supplementary Figure S1).

Of interest, patients bearing missense or truncating mutations in *PRF1* and the granzyme family of genes (*GZMA, GZMB, GZMH, GZMK* and *GZMM*) exhibited higher cytolytic levels in contrast to the *wild-type* tumors, despite the low rate (<2%) of these somatic mutations (Figure 1C and D). Consistent with our previous findings (12), these results show that CYT-high colon tumors are more frequently hypermutated and that those having somatic mutations in granzyme and perforin genes exhibit higher levels of immune CYT.

Clusters of simultaneous multiple mutations, or kataegis, were previously detected in different types of tumors (36,52,60–62), but their association with different levels of CYT in CRC has not been investigated before. Motivated by our initial findings, we set to explore whether CYT associates with kataegis in these tumors. Overall, we identified 42 kataegic sites in 20 colon tumors, associated with 1280 mutations. To our surprise, the majority of COAD tumors with kataegic sites (14/20, 70%) were CYT-high, whereas none of them was CYT-low (Figure 1E and F and Supplementary Table S1). We also found 13 kataegic sites in four READs (associated with a total of 488 mutations), three of which were CYT-high and one, CYT-low.

Intriguingly, half of the mutations in COAD were C>T transitions (51%), which is in agreement with the concept that kataegis is a consequence of DNA replication over cytidine deamination of resected DNA (38,62). In READ tumors, the rate of C>T mutations was lower (31.76%). In contrast to COAD, READ tumors had a high prevalence of C>A transversions (49%) (Figure 1G), suggesting the existence of different mutational processes between the two cancer types. Additionally, differences in the rate of the various types of mutations were observed between the two cytolytic subgroups; for example C>A rates were higher among CYT-low tumors, whereas C>T rates showed a preference for CYT-high adenocarcinomas (Figure 1G). Of note, we found similar intra-tumoral heterogeneity (MATH scores) between the two cytolytic subgroups of tumors, as stated previously (12), which suggests that the patterns of kataegis that we observed in the genome, can distinguish cytolytically diverse tumors, and are not the result of variabilities in the intra-tumoral heterogeneity.

Taken together, our findings reveal that CYT-high (primarily colon) tumors are enriched in kataegis, being suggestive of an involvement (at some level) of the APOBEC cytidine deaminases in the genome of these tumors.

### Low mutation load in APOBEC-enriched colon tumors

APOBEC-induced mutations are more frequent in solid tumors and primarily associate with C>T transitions in the tCw motif (also known as APOBEC motif) (54). The direct link between APOBECs and kataegis was recently obtained by expressing hyperactive deaminase in yeast cells (39). Recent evidence has linked the over-expression of APOBEC3B with various human cancers, including CRC, highlighting its possible contribution to genomic instability and kataegis (63,64). However, the role of other APOBEC family members has not been previously appreciated in CRC, especially in terms of the tumor's immune CYT.

**Figure 2.**
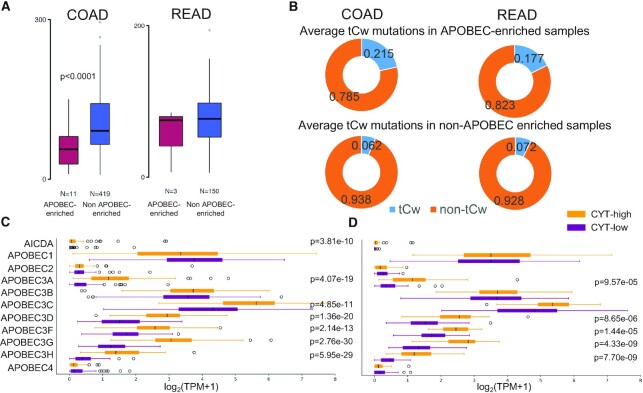
(**A**) Mutation load between APOBEC-enriched and non-APOBEC enriched CRCs. The mutation load was lower in APOBEC-enriched compared to non-APOBEC enriched COAD (but not READ) tumors. (**B**) Average tCw mutations in APOBEC-enriched and non-APOBEC enriched samples. The mutation load had a preference in tCw mutations in APOBEC-enriched tumors. In colon cancer, the average percentage of tCw mutations was 21.5% in APOBEC-enriched and 6.2% in non-APOBEC enriched tumors. Similarly, in rectal cancers, the average percentage of tCw mutations was 17.7% in APOBEC-enriched versus 7.2% in non-APOBEC enriched tumors. (**C** and **D**) Expression levels (log_2_(TPM+1)) of the AICDA/APOBEC family of genes across the two immune cytolytic subgroups in COAD (C) and READ tumors (D). APOBEC3A, -D, -F, -G and -H were significantly overexpressed across cytolytic-high COAD (C) and READ (**D**) tumors. AICDA and APOBEC3C were overexpressed in CYT-high COAD (but not READ) tumors.

Meanwhile, AID is an essential enzyme that generates antibody diversity by regulating class switch recombination, somatic hypermutation and gene conversion (65). AID is also involved in site-specific mutations and the formation of kataegis in B-cell lymphomas (63), whereas the APOBEC3 genes are implicated in genomic mutations in non-B-cell tumors (37,66). To further explore the role of APOBECs in enriched kataegic loci across CRC tumors, we investigated the mutational load in APOBEC-enriched and non-APOBEC enriched CRCs. Notably, the mutation load was significantly lower in APOBEC-enriched colon tumors and had a preference to tCw mutations, providing further evidence of its active role in these cancers (Figure 2A and B). Motivated by these observations, we explored the expression of AID and all APOBEC family members, to address whether they correlate with the observed extent of APOBEC-mediated mutagenesis. We also compared the AID/APOBEC gene expression levels between the two cytolytic subgroups in COAD and READ tumors. Our analysis revealed that APOBECs -1, -3B and -3C are highly activated in CRC. It also showed intense activity of AID (AICDA), APOBEC3A, APOBEC3C, APOBEC3D, APOBEC3F, APOBEC3G and APOBEC3H within CYT-high COAD, and of APOBEC3A, APOBEC3D, APOBEC3F, APOBEC3G and APOBEC3H in CYT-high READ (Figure 2C and D), suggesting that inflammation within cytolytic high colorectal tumors switches on the intense activity of various APOBEC genes.

### Clusters of chromothripsis are more abundant among cytolytic low COAD tumors

Local chromosome shattering, or chromothripsis, is found in 2–3% of cancers (34) and has been proposed to lead to clusters of chromosomal rearrangements. These, can drive cancer development either by deleting tumor suppressor genes or increasing the CNs of oncogenes.

We have recently shown that CYT-low colon tumors harbor significantly more recurrent somatic CN changes (12). Additionally, in our comutation analysis we observed that hypermutated tumors were broadly devoid of CN alterations, relative to their non-hypermutated counterparts (Figure 1A). This suggests that hypermutation, and therefore high levels of CYT, associates with a lack of CN gains or losses. To investigate this further, we set to explore the chromothripsis events in the two cytolytic subsets in CRC.

**Figure 3.**
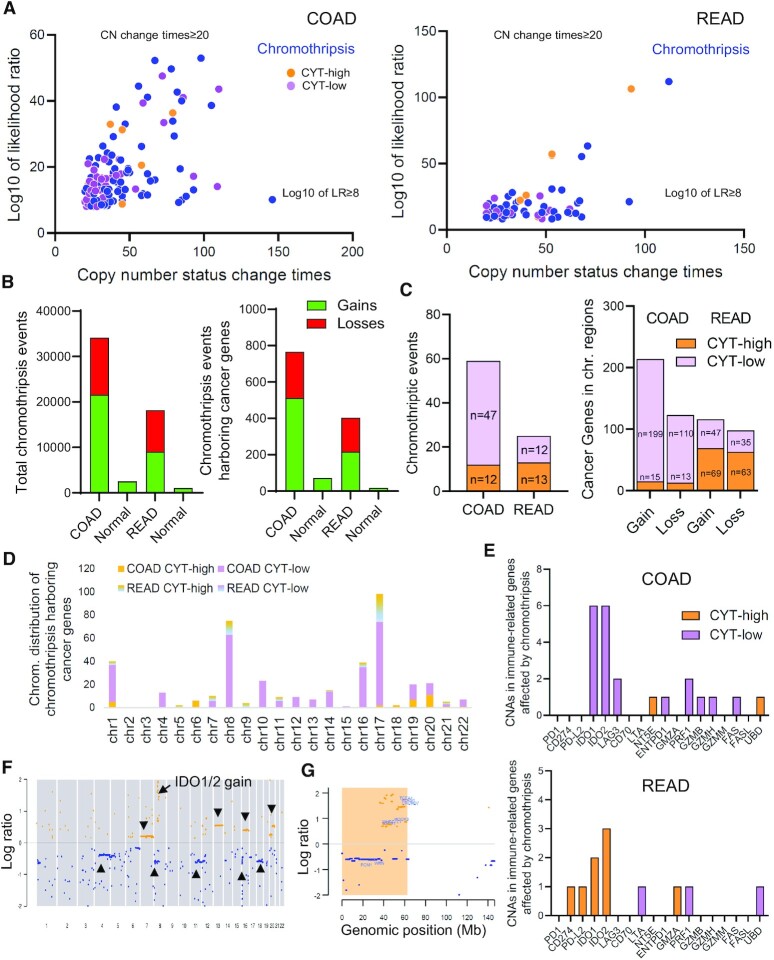
(**A**) Scatter plots of detected chromothripsis events among COAD and READ tumors. The CN status change times are compared against the LR of each event. Each point in the plot represents a chromosome and the closer it is to the top right corner, the more accurately the algorithm can distinguish chromothripsis from non-chromothripsis cases. The criteria used to distinguish chromothripsis were CN change ≥ 20 times, LR (log10) ≥ 8, minimum segment size = 10 kb and signal distance between adjacent segments = 0.3. Chromothriptic events associated with CYT-high and CYT-low colorectal tumors are highlighted in orange and purple, respectively. (**B**) The total number of chromothriptic events (gains and losses), as well as of the chromothriptic events harboring cancer genes, is significantly higher in COAD and READ tumors compared to the normal controls. (**C**) In CYT-low COAD tumors, the number of chromothriptic events was three times higher than that in CYT-high tumors. The number of chromothriptic events across cytolytic subsets in READ was the same. CYT-low COAD tumors contained significantly more cancer genes harbored within chromothriptic regions. The CYT-high READ tumors had chromothriptic regions that harbored more cancer genes compared to CYT-low READ tumors. (**D**) The majority of chromothriptic regions harboring cancer genes, mapped within genomic regions of mainly chromosomes 1, 8 and 17 (199 gains and 110 losses in CYT-low COAD versus 15 gains and 13 losses in CYT-high COAD). (**E**) Representative copy number aberration (CNA) profile of a CYT-low COAD tumor (TCGA_COAD_777396) with chromosome 8 gain, affecting the IDO1 and IDO2 loci. Other gains/losses with a lower log ratio are arrowheaded in chromosomes 4, 7, 8, 11, 13, 16, 18 and 20. (**F**) Chromothripsis plot of this tumor focusing on chromosome 9 shows that the loci *TCEA1, PLAG1, CHCHD7, HOOK3, WHSC1L1, FGFR1* are gained, whereas loci *PCM1, WRN* are lost (highlighted in orange).

Globally, we discovered chromothriptic events of several sizes in 100/976 (10.25%) COAD and 49/318 (15.41%) READ segmentation data, corresponding to 457 COAD and 164 READ tumors, respectively (Figure 3A and Supplementary Table S2). Most individual CRC genomes exhibited more than one chromothriptic-like events (Supplementary Figure S4). The number of detected chromothripsis (and chromothriptic-like) segments was significantly higher among cancerous tissues compared to the blood derived normal samples (controls) with 21 579 versus 2494 gains and 12 551 versus 55 losses in COAD and 9035 versus 998 gains; 9161 versus 41 losses in READ) (Figure 3A). The number of chromothriptic clusters affecting cancer genes was also higher among cancerous tissues (513 versus 71 gains and 253 versus 0 losses in COAD compared to controls; 217 versus 17 gains and 186 versus 0 losses in READ compared to controls). These include *APC, KRAS, BRAF* and *TP53* gains or *FBXW7* losses (*P* < 0.0001, Fisher exact test) (Figure 3B and Supplementary Table S2).

It is intriguing that we found three-times more chromothriptic events in CYT-low compared to CYT-high COAD tumors (47 versus 12); whereas, the number of chromothriptic events was equal between the two cytolytic subgroups in READ (*n* = ∼12) (Figure 3c). CYT-low COAD tumors contained significantly more cancer genes harbored within chromothriptic regions (*TCF12, NF1, ERBB2, JUN, JAK1* and *BCL10*). Additionally, chromothripsis was widely noticed within chromosomes 1, 8, 16 and 17 (199 gains and 110 losses in CYT-low COAD relative to 15 gains and 13 losses in CYT-high COAD) (Figure 3C and F). The same however, was not observed in READ tumors. Overall, in READ there were 47 gains and 35 losses in CYT-low patients relative to 69 gains and 63 losses in CYT-high patients (Figure 3C). Regarding specific cancer-associated genes, the CYT-high subgroup had more CNAs, including gains in *NF1, CDK12, ERBB2, RARA, MDM4* and *MYC*; and losses in *BRCA1, CD274* and *APC*. The majority of the detected chromothriptic regions harboring cancer genes, mapped mainly in chromosomes 1, 8 and 17 (especially across CYT-low tumors) (Figure 3D).

We next envisaged whether chromothriptic events have an impact on immune checkpoints or other immune-related genes across each cytolytic subgroup in CRC. Our analysis revealed that chromothripsis affected immune checkpoint loci in chromosomes 8 (*IDO1* and *IDO2* gain) (Figure 3E and F), 10 (*ENTPD1* loss*, PRF1* gain and *FAS* loss), 12 (*LAG3* loss) and 14 (*GZMB* and *GZMH* loss) in CYT-low COAD tumors. Other loci were also gained or lost in each chromosome. For example, in chromosome 8, *TCEA1, PLAG1, CHCHD7, HOOK3, WHSC1L1* and *FGFR1* were gained; whereas *PCM1* and *WRN* were lost (Figure 3G). In CYT-high COAD tumors on the other hand, chromothripsis did not affect immune-related loci, other than one gain that we noticed in NT5E (CD73) and one loss in UBD, both of which are found in chromosome 6.

Chromothriptic events among CYT-high READ tumors involved losses in chromosome 9 affecting *CD274, PDCD1LG2*, and in chromosome 5, affecting *GZMA* and *GZMK*. The loci for *IDO1/2* (chromosome 8) were either lost or gained in different CYT-high READ tumors. Other immune-related genes were also affected in CYT-low READ, in chromosomes 6 (*LTA* and *UBD* gain), 10 (*PRF1* loss) and 19 (*NKG7* gain) (Figure 3E and Supplementary Table S2). Taken together, our data show that chromothripsis affects more broadly cytolytic low colon tumors.

### Mutational signatures within cytolytic subsets in CRC

Mutational processes from different aetiologies are operative during the course of cancer development and leave particular imprints in the cancer genome, called mutational signatures (52). The record of the accumulated DNA mutations is determined by the intensity and duration of all active mutational processes (36). Genetic mutability can emerge in the form of different classes of mutations, such us single or DBS, short insertions and deletions (indels), CN alterations or structural variations.

**Figure 4.**
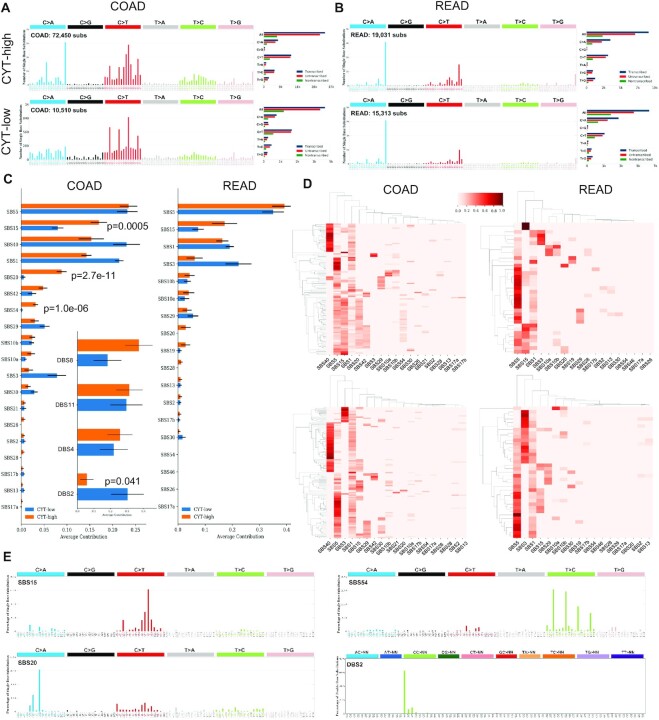
Mutational spectra generated from combinations of single base (SBS, left) signatures in the two cytolytic subgroups in COAD (**A**) and READ (**B**) tumors. The barplots in the middle show the transcriptional strand bias for SBS in each cytolytic subgroup of COAD and READ tumors. Each signature is presented according to the 96 substitution classification defined by the substitution class and sequence context immediately 3′ and 5′ to the mutated base. The probability bars for the six types of substitutions are displayed in different colors. The mutation types are on the horizontal axes, while vertical axes depict the percentage of mutations attributed to a specific mutation type. All mutational signatures are displayed based on the trinucleotide frequency of the human genome. (**C**) Average contribution of each of the (SBS and DBS) mutational signature in the two cytolytic subsets in COAD and READ tumors. (**D**) Two-way hierarchical clusters (HCl) of CYT-high (upper HCl) and CYT-low (lower HCl) COAD and READ tumors. (**E**) SBS15, SBS20, SBS54 and DBS2 were the mutational signatures whose average contribution differed significantly between CYT-high and CYT-low tumors.

We extracted each patient's mutational signatures, and compared them against validated signatures in COSMIC (v3.1) to assess differences between the two cytolytic subgroups. Intriguingly, we found an ∼8-fold higher number of SBSs in CYT-high compared to CYT low COAD tumors (72 450 versus 10 510 SBSs) (Figure 4A). The mutational signatures exhibiting the highest contribution were SBS5 (clock-like, age-related with transcriptional strand bias for T>C substitutions at ATN context), SBS15 (dMMR), SBS40 (unknown aetiology, correlated with age) and SBS1 (also clock-like, associated with spontaneous or enzymatic deamination of 5-methylC to T which generates G:T mismatches in double stranded DNA). Additionally, the following SBSs contributed to a much lower level: SBS20 (concurrent POLD1 mutations and dMMR), SBS42 (occupational exposure to haloalkanes with transcriptional strand bias of C>A and C>T mutations), SBS54 (possible contamination with germline variants), SBS10a/b (POLE defects) and SBS3 [defective homologous recombination (HR)-based repair]. Of note, most of these signatures were previously unappreciated in COAD. Interestingly, the contribution of SBS15, SBS20 and SBS54 differed significantly between the two cytolytic subsets in COAD tumors (Figure 4C). Our analysis also revealed that COAD tumors that were enriched in SBS40, the aetiology of which is unknown, clustered separately from those having a higher contribution in signatures SBS5, SBS15 and SBS20 (in CYT-high COAD) or signatures SBS5, SBS3 and SBS15 (in CYT-low COAD) (Figure 4D). We also noted that the second clusters contain tumors with a higher level of signatures that are characteristic of dMMR/MSI cancers and with defects in proofreading polymerases δ and ϵ (SBS15, SBS20 and SBS10a/b) (4) (Figure 4D).

Although the total number of doublet-substitutions (DBS) was low, we were able to detect ∼6-fold higher number of DBSs among CYT-high COAD tumors (290 in CYT-high versus 46 in CYT-low) (Supplementary Figure S1). Of these, DBS8 (unknown aetiology), DBS11 (unknown aetiology, possibly related to APOBEC mutagenesis) and DBS4 (unknown aetiology, correlates with age of cancer diagnosis) were the dinucleotide mutation signatures that contributed mostly in both cytolytic subgroups in COAD. Additionally, DBS2 (tobacco smoking and other mutagens, e.g. acetaldehyde) contributed significantly more in CYT-low COAD compared to CYT-high tumors (*P* = 0.041). This mutational signature was previously unappreciated in colon cancer, and exhibits a transcriptional strand bias with more GG>TT mutations than CC>AA on the untranscribed strands of genes, indicative of damage on guanine and repair by TC-NER.

In READ on the other hand, the number of either SBS or DBS mutational signatures did not differ significantly between the two cytolytic subgroups (SBSs, 19 031 versus 15 313; DBSs, 31 versus 22) (Figure 4B and Supplementary Figure S2). The mutational signatures with the highest contribution were SBS5 (clock-signature of unknown aetiology), SBS15 (dMMR), SBS1 (clock-like) and SBS3 (defective HR-DNA damage repair). Four mutational signatures also contributed to a lower extent. These were SBS10a/b (POLE mutations), SBS29 (tobacco chewing with weak transcriptional strand bias for C>A mutations), SBS20 (concurrent POLD1 mutations and dMMR) and SBS19 (unknown aetiology with a transcriptional strand bias of C>T mutations with more mutations of G than C on the untranscribed strands of genes consistent with damage to guanine and repair by TC-NER) (Figure 4C). Interestingly, two mutational signatures appeared to differ between the two cytolytic subgroups in READ tumors. The average contribution of SBS15 was higher in CYT-high tumors and that of SBS3, was higher in CYT-low tumors. However, after Bonferroni correction and due to the small READ sample number, the difference did not reach statistical significance (*P* > 0.05) (Figure 4C). By clustering the cancer samples and mutational signatures, we could also observe some level of mutual exclusivity between SBS3 on the one hand and SBS5 with SBS15 on the other, in both READ cytolytic subgroups (Figure 4D).

Taken together, our analysis highlights the existence of multiple mutational signatures in CRCs the impact of which differs depending on the immune CYT profile of the tumor.

### Cell type fraction analysis, cancer neoantigens and prediction of patients’ response to immune checkpoint inhibition

Although early trials using PD-1/PD-L1 inhibitors have been promising, the response rate is limited to ∼30% of mainly MSI-H metastatic CRC patients (28,29). Therefore, the need to elucidate the mechanisms of resistance to immune checkpoint inhibition and to predict more effective therapeutic markers, is of high necessity (67). Since CYT-high CRCs are enriched in CD8+T cells (12), we assumed that they should have higher immunophenoscores, as a result of an increased immunogenicity, indicating that they should respond better to immunotherapy.

To delineate this, we analyzed the cell type fractions in each immune cytolytic subgroup, and further predicted whether CRC patients are expected to respond differently if they were to be treated with different immune checkpoint inhibitors, as assessed from their immunophenoscores.

**Figure 5.**
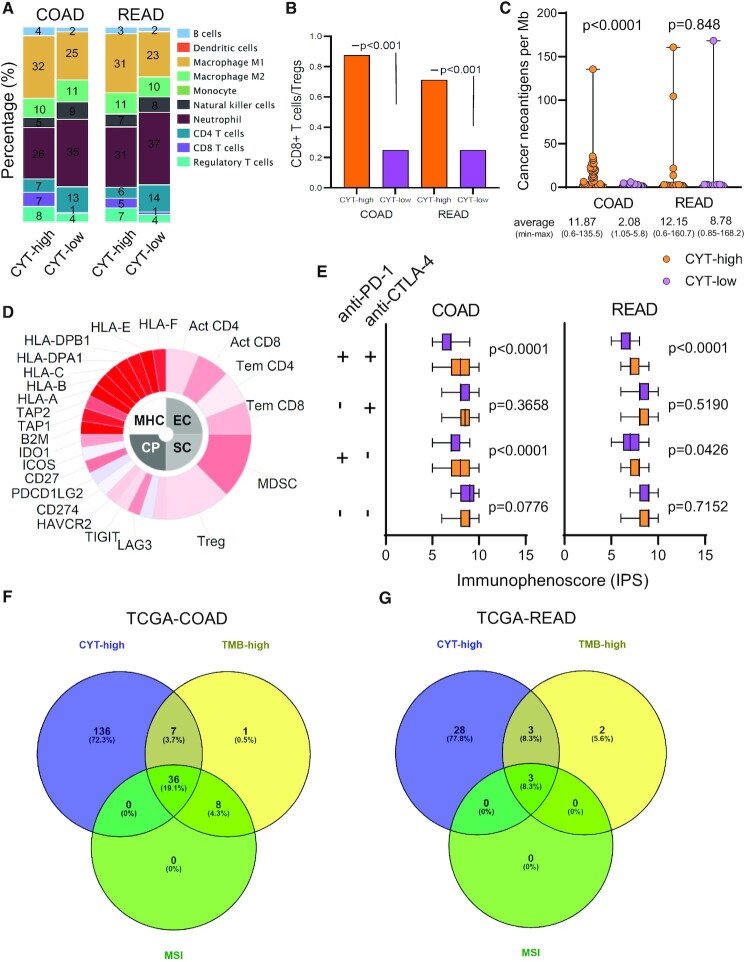
(**A**) Immune cell type fraction analysis across distinct cytolytic subgroups in CRC. CYT-high tumors were significantly enriched in CD8+ T cells and M1 macrophages. On the other hand, CYT-low tumors were enriched in neutrophils and CD4+ T cells. (**B**) Analysis of the CD8+ T cells/regulatory T cells (Tregs) ratio in COAD and READ tumors. The CD8+ T cell/Tregs ratio is significantly higher in CYT-high than in CYT-low tumors (*P*< 0.001). (**C**) Predicted cancer neoantigens in CYT-high and low CRCs. CYT-high COAD tumors had markedly more cancer neoantigens in contrast to CYT-low ones (*P*< 0.0001). (**D**) Indicative immunophenogram, in which the sample-wise *z*-score from gene expression of all cell types included in any of the ten best predictors within each cancer type are color coded and divided into four categories; MHC molecules (MHC), immunomodulators (CP), effector cells (EC) and suppressor cells (SC). The outer part of the wheel includes individual factors; whereas, the inner wheel illustrates the weighted average *z*-scores of the factors included in the particular category. The immunophenoscores (IPS) were calculated on a 0–10 scale based on the expression of the representative genes of the immunophenogram. Sample wise *z*-scores were positively weighted according to stimulatory cells and negatively weighted according to inhibitory cells and averaged. *Z*-scores ≥ 3 were designated as IPS10 and *z*-scores ≤ 0 as IPS0. (**E**) The boxplots indicate the average immunophenoscore values (IPS) across the two cytolytic subgroups in COAD and READ tumors. Overall, CYT-high tumors that could be treated with combined anti-PD-1 and anti-CTLA-4 checkpoint blockade or with anti-PD-1 alone, had significantly higher IPS, which is indicative of a better response to these immunotherapies. (i) anti-CTLA-4 (−), anti-PD-1 (−): patients who would not receive immunotherapy with either anti-CTLA-4 or anti-PD-1 blockade; (ii) anti-CTLA-4 (−), anti-PD-1 (+): patients who would receive immunotherapy with anti-PD-1 alone but not anti-CTLA-4; (iii) anti-CTLA-4 (+), anti-PD-1 (−): patients who would receive immunotherapy with anti-CTLA-4 alone, but not anti-PD-1; (iv) anti-CTLA-4 (+), anti-PD-1(+): patients who would receive combination immune checkpoint inhibition therapy. (**F** and **G**) The Venn diagrams show that the majority of the CYT-high colorectal tumors (COAD, 136/179, 75.97% and READ, 28/34, 82.35%) in the Colorectal Adenocarcinoma TCGA PanCancer dataset, were neither TMB-high nor MSI, suggesting that CYT-high CRC patients may benefit from immune checkpoint blockade.

Our analysis showed a significant enrichment of M1 macrophages, CD8+ T cells and Tregs in the TME of CYT-high tumors. Contrariwise, we found significantly higher levels of neutrophils and CD4+ T cells in the TME of CYT-low samples (Figure 5A). Additionally, the CD8+ T cells/Tregs ratio was significantly higher among CYT-high CRCs (Figure 5B). These findings are consistent with our recent observations in MSI-high CRCs, which contain similarly higher percentages of CD8+ T cells and M1 macrophages, but also fewer neutrophils, as contrasted with MSS and MSI-low tumors (12,45). Additionally, the cell type fraction profile of CYT-high CRCs matches that of CMS2 cancers (i.e. hypermutated, MSI+ with a strong immune activation) (8).

Overall, we examined 30 562 predicted cancer neoantigens across 5540 genes (Supplementary Table S2). CYT-high COAD had markedly higher load of cancer neoantigens (mutations per Mb) compared to CYT-low tumors [average (min-max); CYT-high versus CYT-low, 11.87 (0.6–135.51) versus 2.08 (1.05–5.88)]; In READ, the load of neoantigens did not differ significantly between the two cytolytic subsets [average (min-max); CYT-high versus CYT-low, 12.15 (0.57–160.73) versus 8.778 (0.85–168.16)] (Figure 5C).

Next, we investigated how differences in the IPS score between patients would reflect on a hypothetical treatment with immune checkpoint inhibitors. Importantly, our analysis revealed significantly higher IPS scores (*P* < 0.0001) in CYT-high COAD patients who would either receive combination blockade for CTLA-4 and PD-1 (‘anti-CTLA-4 (+), anti-PD-1 (+)’), or for PD-1 alone (‘anti-CTLA-4 (−), anti-PD-1 (+)’), compared to their CYT-low counterparts (*P* < 0.0001, Figure 5D and E). It is intriguing that the IPS scores were significantly higher in CYT-high tumors, irrespective of their MSI status (Supplementary Figure S3).

On the other hand, patients expected to be treated with an anti-CTLA-4 monoclonal antibody, but not anti-PD-1, or those who would not receive immune checkpoint inhibition at all, did not exhibit different IPS scores. This suggests that high cytolytic levels are expected to play an important role mainly in PD-1 blockade, or when this is combined with CTLA-4 inhibition, and that CYT-high COAD patients are anticipated to have a clinical benefit. In READ on the other hand, it seems that only an anti-PD-1/anti-CTLA-4 combinatory immune checkpoint inhibition should significantly benefit CYT-high patients (*P* < 0.0001) (Figure 5E).

To enhance our suggestion that the CYT-high subgroup of CRCs is a previously unappreciated cohort that could benefit from immune checkpoint blockade, we examined the distribution of the tumors across their CYT, tumor mutation burden (TMB) and MSI status, using the Colorectal Adenocarcinoma TCGA PanCancer data. Interestingly, 136 out of the 179 CYT-high colon cancer patients (75.97%) and 28 out of the 34 CYT-high rectum cancer patients (82.35%) were neither TMB-high nor MSI (Figure 5F and G), excluding the possibility that the CYT-high subgroup of tumors is the same as those with a high TMB or MSI status. This provides a good indication that a significant proportion of the CYT-high CRC patients, indeed, belong to a previously unappreciated cohort that could benefit from immune checkpoint inhibition therapy using anti-PD-1 alone or in combination with anti-CTLA-4 monoclonal antibodies.

Taken together, our data delineate differences in the fraction of immune cells in the TME of each cytolytic subgroup in CRC. They also reveal that CYT-high COAD tumors contain a higher load of cancer neoantigens in their genome. Last, they underline the predictive value that immunophenoscores have in CYT-high CRC patients if they were to be treated either with a combination of CTLA-4 and PD-1 blockers, or with PD-1 blockers alone.

## DISCUSSION

Recent data show that intratumoral CYT can predict a cancer's immunity (17). Distinct cytolytic subgroups in CRC are characterized by different genomic and transcriptomic events (12,18). As such, insights on how different types of lesions in the CRC genome associate with immune cytolytic subgroups can help us provide more effective immunotherapies for these patients, especially for the pMMR/MSS ones, who are currently unresponsive to immune checkpoint inhibition therapy.

Some somatic mutational processes produce numerous mutational clusters in a single catastrophic event, which substantially reconfigure the genome (64). Kataegis and chromothripsis are two such processes that have been extensively investigated lately (34,36,43,64,68–72). In our study, we questioned whether kataegis and chromothripsis impact immune checkpoints or other immune-related genes across immune cytolytically diverse CRCs. In addition, we explored differences in the mutational signatures in the context of CYT. Our results reveal that CYT-high colon tumors are hypermutated and enriched in kataegis. Hypermutation among cytolytic high CRC has been previously reported (12,73). However, we provide a further link between mutations in GZMA and perforins and high CYT in colon cancer. We also provide evidence that CYT-high colon tumors are enriched in kataegis, harboring mainly C>T mutations, compared to CYT-low tumors. Additionally, we found differences in the rate of the various mutational types between the two cytolytic subgroups in CRC.

We discovered a significantly lower mutational load across APOBEC-enriched colon tumors with a preference in the tCw APOBEC motif. This corroborates previous reports that CRCs do not display a strong APOBEC pattern compared to other tumors (52,54). However, our data show that certain APOBECs (e.g. APOBEC3A, APOBEC3C, APOBEC3D, etc.) are highly expressed in CYT-high colon tumors, providing evidence of their involvement in them. The association of APOBEC1 in human gastrointestinal tumors and colon cancer-derived cell lines was initially reported some 20 years ago (74,75). Since then, other members of the APOBEC family have also been implicated in the disease. The overexpression of APOBEC3G was previously detected in CRC and shown to predict hepatic metastasis (76,77). The Pan-cancer analysis of whole genomes (PCAWG) study recently showed that the APOBEC signature accounts for the majority of kataegis events and that it correlates with the expression of APOBEC3B, the number of somatic structural variations and age at diagnosis (64).

We also found a higher number of chromothriptic clusters in CYT-low COAD tumors. As this is suggestive of the existence of further complex events in the genome of these tumors, we explored whether chromothriptic events affect cancer genes and immune-related loci. Notably, we found that chromothripsis affects cancer genes including *APC*, *KRAS*, *BRAF*, *TP53* and *FBXW7*, but also several immune checkpoints or immune-related genes, like *CD274*, *ENTPD1*, *PRF1*, *FAS*, *LAG3, IDO1* and *IDO2*.

Our findings provide evidence for the existence of mutational signature differences between cytolytic subgroups in CRCs. In accordance to the recent data from PCAWG (30), we showed that the most prevalent mutational signatures in CRC are SBS5, SBS15, SBS40 and SBS1; but we provide further evidence that other, previously unappreciated mutational signatures, also contribute at a lower extent. It was also intriguing that the contribution of SBS15, SBS20 and SBS54 differed significantly between the two cytolytic subsets in COAD tumors, as well as that tumors enriched in SBS40 clustered separately from those having a higher contribution in signatures SBS5, SBS15 and SBS20 in CYT-high COAD or in SBS5, SBS3 and SBS15 in CYT-low COAD. Previous studies have also reported the involvement of mutational signatures related with age, dMMR/MSI or defects in polymerase ϵ (4,78). However, we extend this knowledge by providing further association with defects in polymerase δ (SBS20), the signature that has been associated with occupational exposure to haloalkanes (SBS42) and defects in the HR repair pathway (SBS3). The identification of mutational signatures responsible for the specific mutational patterns was also proposed to have significant therapeutic implications (79,80). For example, the development of DNA repair deficiency biomarkers is critical to the implementation of therapeutic targeting of repair-deficient tumors, using either DNA damaging agents or immunotherapy for the personalization of cancer therapy (79). Genetic defects in HR-based repair pathway were recently reported in CRC by others (80,81) and HR deficiency was proposed to occur in cells with no detectable BRCA1/BRCA2 mutations but exhibiting BRCA-like phenotypes. DNA repair-targeting therapies, such as ATR and CHK1 inhibitors, were proposed for use in combination with current genotoxic chemotherapies in CRC to further improve therapy response (81). The significance of the contribution of the SBS3 mutation is reflected by the recent finding of a subset of vulnerable to poly(ADP)-ribose polymerase (PARP) inhibition colorectal tumors, which could benefit from the PARP inhibitor olaparib (80).

Immune checkpoint inhibitors targeting PD-1 (pembrolizumab and nivolumab), PD-L1 (atezolizumab and durvalumab) or CTLA-4 (tremelimumab and ipilimumab) have been granted FDA approval for metastatic CRC patients with chemorefractory dMMR-MSI-H, which represent just an ∼5% among all metastatic cases (25,82,83). Nevertheless, not all dMMR-MSI-H tumors respond well to these immunotherapies, with a significant proportion exhibiting resistance to them. In addition, pMMR–MSI-L tumors are largely unresponsive to current immune checkpoint inhibition therapies, and therefore, it is necessary to develop suitable therapies for these patients, as well. A major question is why some patients do not respond to single agent immune checkpoint inhibition, and there is a great concern of how these patients can overcome resistance and harness immunotherapy.

To this end, we explored the fractions of various immune cells and cancer neoantigens within cytolytic subgroups in CRC, and predicted which of them would respond better to a hypothetical treatment with CTLA-4 and PD-1 blockers, either alone or in combination. Regarding the first aspect, our data corroborate an enrichment of M1 macrophages, CD8+ T cells and Tregs in CYT-high tumors, which are also characterized by a higher cancer neoantigen burden. Previous studies attributed a good prognosis to infiltration of M1 macrophages (84), NK cells and CD8+ T cells (85). Overall, a subset of CRC patients shows a high degree of immune cell infiltration in their TME (86), whereas others exhibit a high presence of mesenchymal stromal cells in it (87), both of which demonstrate relevant cross-talk with the tumor.

Predicting tumor responses to PD-1 blockade and selecting the optimal patient population remains a major challenge. The dMMR/MSI-high patients are a target population for PD-1 blockade; however, they are only a subset of the total patients. We analyzed the IPS scores of CYT-high and low CRC patients, and found that irrespective of their MMR/MSI status, CYT-high colon patients could benefit more from anti-PD1 treatment alone or in combination with anti-CTLA-4, in contrast to CYT-low patients. Therefore, it seems that the pre-existing antitumor immunity in CRC patients can predict their response to immune checkpoint inhibition therapies. The combination of different immune checkpoint inhibitors is a promising approach that is currently under investigation. For example, combination of nivolumab with ipilimumab was evaluated in the CheckMate 142 (NCT02060188) trial, and 33% of the patients achieved an objective response, whereas 52% of them achieved stable disease (28). Updated results of CheckMate 142 in the complete cohort of dMMR/MSI-H CRC patients treated with combination of nivolumab and ipilimumab, demonstrated enhanced clinical benefit and manageable safety, representing a new standard of care in these patients (88).

To sum up, our data highlight new links between distinct genomic events, such as kataegis and chromothripsis and the activation of an antitumor immune response in cytolytic subgroups in CRC. Our findings also reveal new insights regarding differences in the mutational signatures between cytolytic subsets in CRC. Finally, we provide evidence that the cytolytic index, irrespective of the MMR/MSI status of the tumor, should be taken into consideration when selecting patients for immune-checkpoint inhibition therapy.

## DATA AVAILABILITY

All data generated or analyzed during this study are included in this published article and its supplementary information files.

## Supplementary Material

zcab005_Supplemental_Files
