# Supplementary material for: Molecular correlates of immune cytolytic subgroups in colorectal cancer by integrated genomics analysis
Source: NAR Cancer. 2021 Mar 2;3(1):zcab005. doi: 10.1093/narcan/zcab005 (PMC8210146; doi:10.1093/narcan/zcab005)
Supplement: zcab005_Supplemental_Files [file zcab005_supplemental_files.zip › Supplementary data.pdf]

Supplementary data

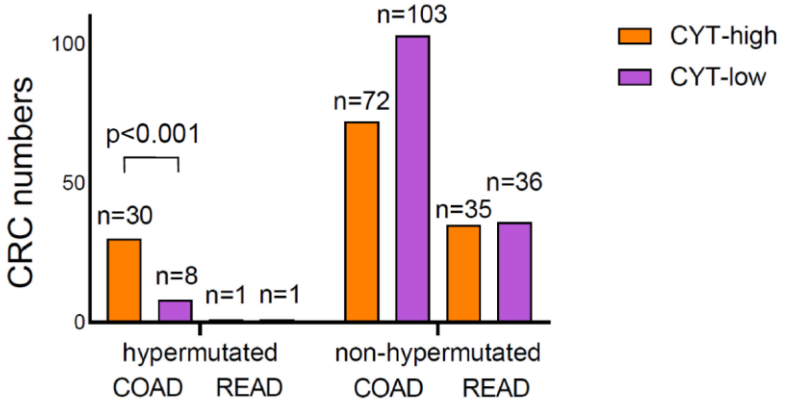

Figure S1

Figure S1. Frequency of hypermutated and non-hypermutated COAD and READ tumours across each cytolytic subgroup. Thirty CYT-high COAD tumours were hypermutated (30/102, 29.41%), whereas the corresponding percentage of hypermutation was much lower in CYT-low COAD tumours (8/111, 7.2%) (p-value<0.001, Fisher's exact test). On the other hand, only one CYT-high READ tumour was hypermutated in CYT-high (1/36, 2.77%) and CYT-low (1/37, 2.70%) READ tumours, respectively.

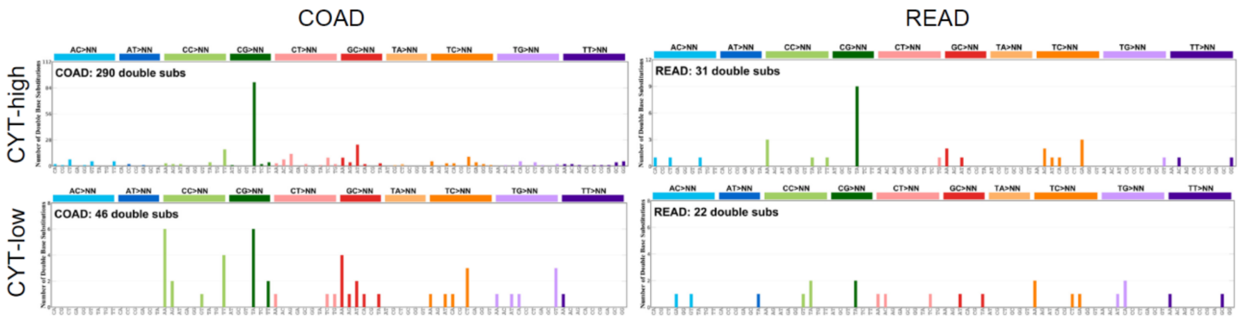

Figure S2

Figure S2. Mutational spectra generated from combinations of doublet base signatures (DBS) in the two cytolytic subgroups in COAD and READ tumours. The barplots in the middle show the transcriptional strand bias for DBS in each cytolytic subgroup of COAD and READ tumours.

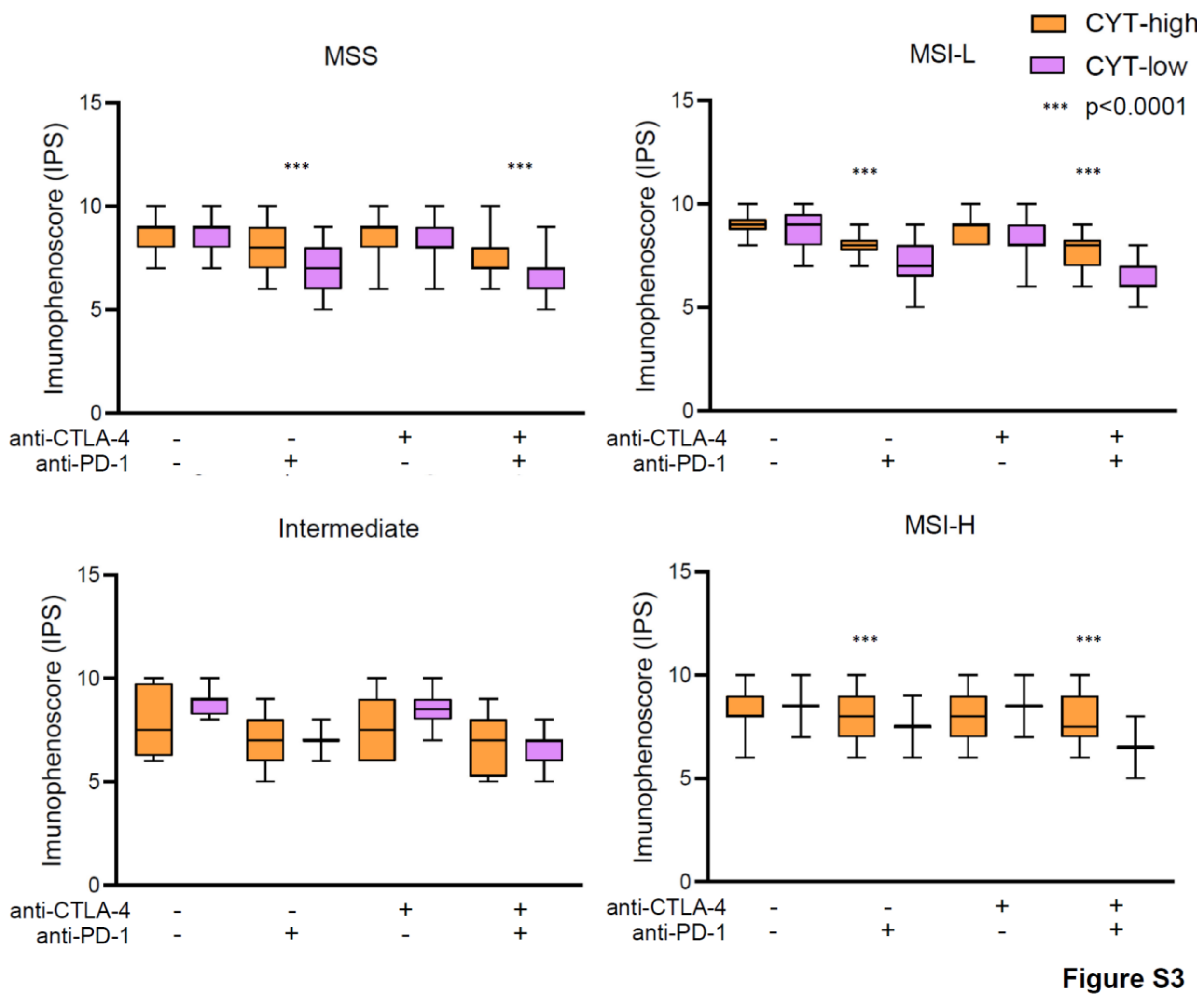

Figure S3. The boxplots indicate the average immunophenoscore values (IPS) across each cytolytic subgroup in COAD and READ cancers, given their microsatellite instability status (Microsatellite stable tumours, MSS; Microsatellite instability-low tumours, MSI-low; Intermediate microsatellite instability; Microsatellite instability-high tumours, MSI-H). Overall, CYT-high tumours that could be treated with combined anti-PD-1 and anti-CTLA-4 checkpoint blockade or with anti-PD-1 alone, had significantly higher IPS irrespective of their MSI status, which is indicative of a better response to these immunotherapies. (i) anti-CTLA-4 (-), anti-PD-1 (-): patients who would not receive immunotherapy, either with anti-CTLA-4 or anti-PD-1 blockade; (ii) anti-CTLA-4 (-), anti-PD-1 (+): patients who would receive immunotherapy with anti-PD-1 alone and not anti-CTLA-4; (iii) anti-CTLA-4 (+), anti-PD-1 (-): patients who would receive immunotherapy with anti-CTLA-4 alone, but not anti-PD-1; (iv) anti-CTLA-4 (+), anti-PD-1(+): patients who would receive combination immune checkpoint inhibition therapy. ***, p-value<0.0001.

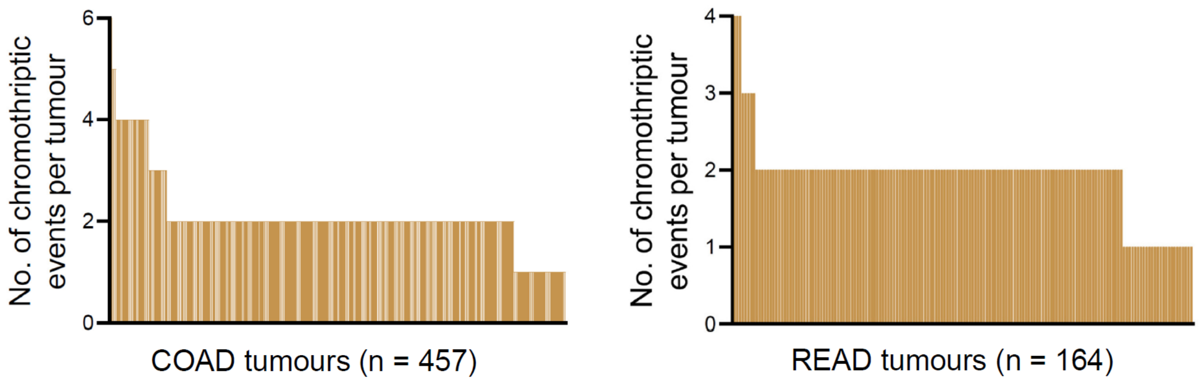

Figure S4

Figure S4. Most individual colorectal cancer genomes exhibit more than one chromothriptic events.

Table S1. Kataegic events detected across different cytolytic subgroups in colon (COAD) and rectum (READ) adenocarcinoma samples.

Table S2. Detailed information on the chromothriptic analysis and the cancer neoantigens per immune cytolytic subgroup in colorectal cancer.
